# Observations on the Nature, Origin, and Treatment of Puerperal Fever, Founded on Cases Which Occurred Epidemically at Plymouth, in the Autumn of 1831; and on a Comparative View of Concurrent Cases of Abdominal Inflammation in the Puerperal State, the Pregnant State, and after a Miscarriage; and of Cases in Women Not Pregnant; of Abdominal Inflammation Occurring in Various Diseases Then Prevalent ; E. G., Synochus, Cholera, Erysipelas, and a Peculiar External Gangrenous Affection; and on a View of Peritoneal Inflammation in the Male Sex

**Published:** 1845-05

**Authors:** Edward Blackmore

**Affiliations:** Edin.


### RECORD OF MEDICAL SCIENCE.

### OBSERVATIONS ON THE NATURE, ORIGIN, AND TREATMENT OF PUERPERAL FEVER.

*Founded* (a) *on Cases which occurred Epidemically at Plymouth, in the Autumn of* 1831*;* (b) *and on a Comparative View of Concurrent Cases of abdominal inflammation in the Puerperal State, the Pregnant State, and after a Miscarriage;* (c) *and of Cases in Women not Pregnant;* (d) *of Abdominal Inflammation occurring in various Diseases then prevalent;* e.g., *Synochus, Cholera, Erysipelas, and a peculiar external Gangrenous Affection; and* (e) *on a View of Peritoneal Inflammation in the Male Sex.*

BY EDWARD BLACKMORE, M. D., EDIN.

The facts and reasonings in this paper were communicated, substantially, to the Bath Medical Society, in the session of 1833. The cases which have come under my notice may be interesting to those practitioners whose attention has been excited by the valuable papers on this most important subject, which have appeared in the *Provincial Medical and Surgical Journal.*

The most impressive view of this disease will be obtained from a perusal of the cases themselves*, and by observing the contrast or resemblance to the puerperal fever, exhibited in the character of the associate diseases. The leading general symptoms of the specific affection were, shivering, attended by pain in the abdomen, a rapid pulse, and quick breathing; in some cases, vomiting; in others, a purging, quickly succeeded by serous effusion into the peritoneum, when this membrane had been the seat of extensive inflammation; and a rapid sinking of the vital powers.

The phenomena of the cases suggest the following deductions:—

(1.) That puerperal fever is a specific affection, which, when occurring either epidemically or sporadically, presents itself under *two forms,* which constitute merely *varieties* of the disease, and not specifically different maladies. These forms may be designated the *synochal,* sthenic, or more benign; and the *typhoid,* asthenic, putrid, or more malignant. The general form of the puerperal fever at any period, usually resembles that of other febrile diseases, particularly synochus, and various exanthemata, and also of some purely local inflammations, which prevail at the time in the same place; and the analogies then observable among all these affections, strongly illustrate the character of each other.

The disease in both varieties is of a *malignant* nature, in comparison of common fevers and inflammation; it assumes a more asthenic tic character in hospitals, low marshy situations, and in a variable autumnal season.

(2.) Disorder in the system, signified by an indescribable uneasiness, or change in the nervous feelings, a quickened pulse, and dis-

* Published by the Author in previous numbers of the Provincial Med. and Surg. Journal.—Ed.

ordered breathing, generally precedes the *manifestation* of the local affection, although to an inattentive observer these two elements may seem to begin and go on with an equal step.

(3.) An extensive and severe abdominal affection is an essential part, and a great source of the danger, of puerperal fever. This local affection has *various seats;* in some cases, the *peritoneum* of the intestines is chiefly affected; in others, that of the *stomach*, or of the *uterus* and its appendages. In all the cases which have come under my observation, the parietal or the visceral serous membrane has been more or less the seat of disease. Disease in the *mucous* membrane of the uterus, intestines, and stomach, forms occasional complications with the peritoneal affection, and severely aggravates the danger of the case; and sometimes this membrane is the original seat of the abdominal affection. In a majority of the cases, the *rapid extension* of the inflammation, whatever be its seat, is a distinguishing character of the local disease.

In some cases, particularly of the typhoid form, besides the former organic lesions, we see a *softened* state of the *liver* and *spleen*, gangrene or fœtid suppuration in the *uterus* and *ovaries*, and a softened or gangrenous state of the *lungs*.

(4.) The specific character of the disease is not derived from an affection of any one organ or texture, nor from any particular morbid state of the abdominal viscera. The character of the constitutional affection, in any particular case, the sympathetic or secondary fever, will, indeed, vary with the nature of the local disease as to its erythematic or suppurative or gangrenous conditions; but in genuine puerperal fever, the primary fever and the local element are equally the pure results of its specific exciting cause. The malignity of the disease, moreover, is not the effect of uterine *phlebitis* and absorption of pus from the veins; nor even of *gangrene* of the viscera. The cases have shown an equally rapid course, in which vascularity and effusion into the peritoneum were the only morbid appearances.

The *degree* of the local affection is variable; in some few cases forming the minor element of the disease; practically, however, it is always an important part of it, and a great source of danger; in its symptoms, and in its progress, it is remarkably different from those of abdominal inflammation from other exciting causes.

(5.) The malignity of the disease is not more mysterious than that which occasionally marks erysipelas, the other exanthemata, and petechial typhus; and, from the cases which I have detailed, it appears to be analogous to these diseases when extensively prevalent and fatal. This malignity is characterised by the severity of the early rigor and shiverings, by the early acceleration of the pulse, and of the breathing, by the intensity of the pain, or by a *want of correspondence* in the degree of the pain with the other alarming symptoms, and by the rapid sinking of the vital powers. The symptoms of a severe affection of the nervous system, so often seen in bad cases of synochus or typhus, were absent in the fatal cases of the puerperal disease that came under my notice.

The malignity is also manifested by the *rapid extension* of erythematic inflammation over the serous and mucous membranes of the abdominal and pelvic viscera, and its attendant serous effusion; and by a suppurative or gangrenous affection of the lungs, the liver, the spleen, and of the subcutaneous and inter-muscular cellular tissue of the trunk and limbs.

The cases which I shall submit to the attention of the Association, seem to demonstrate, that *puerperal fever is not a pure local inflammation.* It is *something more* than *erythematic peritonitis* or *metritis,* in puerperal women; which, in a scrofulous constitution, does exhibit *analogous* phenomena; as do also abdominal inflammations in other subjects when puerperal fever is prevalent.

It is not a pure fever, of which abdominal inflammation is an incidental accompaniment, and which is strikingly exemplified in case 14.

It presents the most remarkable analogies to the *pyrectic exanthemata,* particularly to idiopathic *erysipelas;* (see cases 15, 16, 17, 20;) and from its co-existence with this disease, and their probable community of origin, I should regard it as essentially a puerperal *erysipelatous fever.* Erysipelas with puerperal fever, is known to prevail epidemically, and to exhibit analogous varieties of the more fatal character. The contrast to this puerperal disease, presented by the cases in section 3, and the *resemblance* of the cases in section 4, (see cases 15, 16, 17, 20), strongly confirm this view of the pathology of the fever, and offer a solution of the difficulty in which the pathology of the subject has been involved; and if erysipelas be regarded as essentially a *cutaneous* eruption, attendant on a primary fever, from a specific poison, (although I could adduce strong direct evidence of *internal* erysipelas, and we have the evidence of analogy in scarlatina maligna, affecting the throat and not the skin,) then I would propose to add puerperal fever to the usual enumeration of the species of the exanthemata, defining it "*A contagious disease,* affecting the individual *commonly but once, beginning with a fever, and, after a variable interval, showing itself in an erythematic inflammation of the serous and mucuous membranes, and parenchyma, of the abdominal and pelvic viscera, in the puerperal state.*" If this puerperal fever is a specific disease, differing from a pure inflammation, and always more or less malignant, it becomes a matter of infinite moment *to recognise the first case of the malady,* which may be the forerunner of a fatal epidemic. Can it be distinguished from another case of abdominal inflammation in the puerperal state? The progress of the case, and the after-death appearances, would certainly serve to distinguish it; but the diagnosis then has come too late for the safety of the patient, and probably also for confining its diffusion. A wholesome suspicion of the peculiar nature of the case may be suggested *by the nature of the diseases then prevailing,* particularly the exanthemata and fevers, if it be not excited by the special symptoms of the case.

It is too true that this important recognition has scarcely ever been arrived at; the early cases of a puerperal fever epidemic have usually come on the practitioner as a thunder-bolt; the state of the patient from the period of delivery to the outburst of deadly symptoms, has appeared to betoken nothing alarming nor unusual; the real character of the disease is only revealed to him by the dire experience of fatal cases. *If he had been anticipating* such a disease, he might have seen some symptoms that should have awakened his vigilance. The facts of the following cases enforce this precept:—Whenever synochus is often attended with peritoneal inflammation; whenever erysipelas has been prevailing; whenever other exanthemata have worn a malignant character; whenever the state of the weather has been unusually variable and unnaturally mild and rainy, be on the watch for puerperal fever!

The *causes of the mortality* of this dire disease may be referred to these heads:—(a) The specific nature of the disease, *e. g.*, its extensiveness, its intrinsically asthenic and putrescent character; the sedative influence on the vital powers of the accumulation of the poison in the system, or of the congested venous blood in the abdominal viscera. (b) Early neglect of remedies; many cases are incurable, unless properly treated within twelve hours after the attack. (c) Improper treatment, *e. g.*, large bleedings, abuse of turpentine and opium. This treatment may, indeed, have been pursued without fault in the practitioner, who can sometimes only learn from painful experience, the real nature and proper remedies of the malady. An examination of one's own cases and others on record, will, however, show that failure has been owing to the treatment *not being modified according to the changeful states of the case*, and the true character of the epidemic. Profuse bleedings, so generally practised in the early cases, and sometimes a more moderate bleeding at a late period of the disease, has evidently, in later epidemics, served only to augment the serous effusion, and hasten a fatal collapse. I cannot, however, admit the paralysing belief, which has been derived from misfortune, that in a large proportion of cases all treatment is useless from the time when the medical man is first called in. I am convinced that some patients might have been saved *deo favente*, if the practitioner could have seen the case *unintermittingly*, or had had a judicious assistant on the watch during his absence. More will be said on these points below, under the head of treatment. (d) The *antecedent state of the patient* has sometimes made the attack fatal—*e. g.*, a full-blooded irritable state of the constitution, or debility with poverty of the blood, or an indefinable unhealthiness connected with a sickly season, or a crowding in lying-in hospitals, or a stomach complaint, or disease of the ovaries. See case 2.

The *origin* of this malady is a subject not less important than its pathology. *That it is contagious* in each of its varieties, sthenic or putrid, is beyond dispute. *The source of the infection*, in the first case of the epidemic, *and the principal means of its subsequent diffusion*, are matters of the utmost consequence to be ascertained. The facts bearing on this question, in the epidemic to which this paper refers, are these:—The case first narrated, was the second fatal case among *eight* or more examples of puerperal inflammation, which had occurred to one accoucheur in a fortnight; and in the subsequent fortnight, he had *seven* other cases, of which *three* were fatal; and in another week three or more, and two deaths; and after this he had other cases, which were saved. I ascertained that he had at least *eighteen* cases in rapid succession, and eight of them fatal, the majority of which occurred when no similar cases were heard of in the practice of other accoucheurs. Before, however, the disease had disappeared from the list of this gentleman, who is now deceased, a fatal case occurred to a practitioner, and then others in quick succession; and concurrently with these, similar cases happened to a third accoucheur, among the higher as well as the lower classes, and in all parts of the town! No communication was ascertained betwixt these three sections of cases; none of these medical men had seen the cases of the others; *and the accoucheur was the only known means of communication among his own cases.*

*Whence sprung the first of these cases?* The accoucheur had a large practice among the middle and lower ranks of the population, and had been in constant communication with cases of erysipelas and fever; and as these diseases were prevalent in those parts of the town in which the puerperal fever cases were met with, it is at least highly probable that some of the puerperal patients and their nurses had been in communication with the subjects of those diseases. So far as the evidence goes, it tends to establish the conclusion that the poison of erysipelas was the origin of the puerperal fever. Not that I believe there was, or ever is, necessarily, a direct innoculation of the poison, by the hand of the medical man. I do not believe that the matter of a healthy abscess, with surrounding erythema, a common instance of sporadic or traumatic erysipelas, could, by *itself*, induce puerperal fever. If it could, every accoucheur ought to renounce all surgical practice, and to remain a pure man mid-wife! Admitting that a lying-in woman could suffer at all in this way, she would present a sporadic case of abdominal disease, and no infectious fever would follow. If the poison of erysipelas may have been the origin of the fever, is the accoucheur to oppress himself with remorse, as *if he must have been its vehicle?* This, like the other exanthematous contagions, may assume a gaseous state, *and become a morbific atmospheric influence, and extend itself beyond the range of personal communication;* and I believe that in some instances at least, it had reached the puerperal fever patient, *before she was in child-bed*, or before the practitioner had come near her. Again, before a case of erysipelas has been apparently the origin of this fever, *it has itself been epidemic*, and this fact involves the existence of an atmospheric influence, which may operate apart from personal communication, either immediate or mediate, with the sick. The medical man, then, is not necessarily the *originator* of puerperal fever.

*Is he the principal means of its diffusion?* The cases as we have seen, are for a time confined to one man's patients—the cases occur in rapid and even in *direct* succession—he retires from practice, and on his return, he meets with no other cases. These strong facts strike his conscience, and he feels that he never can forgive himself, for not having retired after the first unequivocal and fatal case! If his conclusions were well founded, the facts should show that he *inevitably* and universally conveys the contagion; that it cannot be diffused in the atmosphere, and that its laws are different from those of all other febrile contagions. The ascertained facts do not show this; the practitioner may be, and to some extent is, innocent of the direct succession of his cases, *yet it is his duty to act as if he would, without precautions, convey the disease.* Let him change every garment that he has worn in the sick room, wash himself from head to foot, (not omitting his *hair* and his *teeth*,) and remain for some hours in the open air, before going to another lying-in-patient. If, after this, a second case occurs, he must retire, from a regard to his own happiness and respect to public opinion; at least, he must so far limit his midwifery practice, as to enable him to carry out the precautions named, to the fullest extent. Let not this admonition be thought dogmatic or magisterial; the exhortation is not needless. I have known an accoucheur to go from a *post mortem* inspection of a case, directly to another parturition!

The facts, then, appear to be, that puerperal fever is contagious, and that the accoucheur, the nurse and infected apartment, and fomites, have spread the disease; that *it is spread also by an atmospheric influence, which no precaution can always exclude from the lying-in room;* and that the poison of erysipelas, itself also communicable in both those modes, is the same as that of puerperal fever, or convertible into it.

It is, further highly probable that certain natural, but extraordinary, atmospheric conditions must occur with the animal poison, in order that these diseases may become epidemic; these conditions being, so far as they are known, a low barometric, and a high thermometric, and hygrometric quality; warm, damp, variable weather, pregnant, also, with thunder. The facts which favour these views are: that when puerperal fever is prevailing, pregnant, puerperal and mis-carrying women are especially liable to abdominal inflammation, and that of a peculiarly paroxysmal and protracted character. (See cases 4 to 10.) That cases of abdominal inflammation then co-existing, also exhibit a peculiar character. That synochus and typhus is then often attended with abdominal inflammation, which, in its erysipelatous character, and the want of proportions in the symptoms and the morbid appearances, resembles the last affection in puerperal fever; and that various exanthemata, then prevalent, also exhibit more or less of a malignant aspect.

I beg leave to apologise for the aphoristic form of the foregoing remarks. I wish not to *dogmatise* on so difficult a subject, but merely to place the more important points prominently before the attention of practitioners. In a subsequent paper, I shall present notes and illustrations, derived from various authorities, on this most interesting disease.

*The Treatment.*—There are three sources of light on the treatment of a disease ; (a) *empiricism*, or a fair deduction from the unsuccessful and the successful treatment of former cases; (b) the *pathology* of the disease : and (c) *analogy*, the results of the treatment of other co-existing maladies of a similar type.

Before using these sources for estimating the treatment of puerperal fever, it may be proper to offer a remark on the doctrine, that the disease, in its malignant form, is absolutely incurable, that the most prompt and vigorous treatment is unavailing, and that our efforts can only be usefully directed to *prevent* the malady. It is true that it has been frightfully fatal; and that the recorded results of medical practice are to some extent nugatory, as many cases published under the name of puerperal fever were not instances of it, but such as my 4th, 6th, 7th, and 9th cases.

Yet a retrospect of the fatal cases, of each form, (and I mean not to exempt my own,) does show that the mortality has not been absolutely referrible to the malignant nature of the disease. To adduce illustrative evidence of this would be painful or invidious; it may, however, be stated that failure has at times been owing to an employment of the remedies proper only for the state of collapse in the congestive stage, and the converse ; to an *unscientific combination* of various sorts of medicines, thus defeating ourselves *propria manu ;* to transferring the fruits of experience gained in one epidemic *indiscriminately* to another of a different character, and to *misapplying* to cases in private practice the experience gained in an hospital. Common sagacity teaches us that every epidemic, and *each case*, should be treated according to its particular nature. With respect to *each case*, the *textures* and the organs affected should *modify* the treatment. In one of my cases, the death might have been owing to a disregard of the *gastric mucous membranes ;* in another case, in which *previous* disease had laid the foundation of grangrenous disorganization of the ovaries, the treatment was such as could only succeed in a pure phlegmonous inflammation.

As to the particular remedies :—(a) *Blood-letting.* In the synochal form of the fever, and in the early state, when the previous health has been good, and the labour unattended with flooding, and when the type of other prevailing diseases is that of inflammatory fever, or phlegmonous inflammation, no reasonable doubt can exist on the propriety of a *general* bleeding.

In many coincident cases of peritoneal inflammation, in puerperal and pregnant women, bleeding was unquestionably the remedy that saved the patients. Case 6 shows the sad consequence of its omission, and Case 7, the happy effect of its timely use. The symptoms, and the morbid appearances, in the puerperal fever cases, showed intense erythematic inflammation. It would be contrary to all just theory and experience to suppose that such a disease could subside without the loss of blood, or that general bleeding, wisely practised as to its seasonableness and quantity, could do harm. Yet experience and pathology, and analogy, combine to show that bleeding is not to be performed, even in the less malignant cases of the fever, as it is successfully done in sthenic phlegmonous inflammation. In the cases I have narrated, large bleedings did not save the patients; probably in two of these it favoured the peritoneal effusion. The analogical evidence from the erysipelatous and gangrenous affection (cases 16, 17, 18, and 20,) is conclusive on this point. In the cases where bleeding is proper, it certainly should not be carried to syncope, nor performed in the erect posture. A moderate general bleeding, followed instantly by leeches to the seat of pain, or to the vulva and groins, or a cupping glass on the top of each thigh, is preferable to a large abstraction of blood from the arms. And the combination of small, general and local bleedings, is better than thirty-six leeches on the belly, to impress the system. By this means, *done early*, the accoucheur, who lost so many patients in 1831, saved the remainder; although the malignity of the epidemic had certainly not then exhausted itself.

The decision on the treatment will often be more difficult than in the case I have mentioned: *e. g.* ought general bleeding to be performed when the case is of the asthenic typhoid form, as in my second case; the previous health infirm, symptoms of disease in the uterine appendages pre-existing, which make it likely that disorganization will quickly follow; when, also, the pulse is very rapid, and when the state of the breathing shows that the lungs, or the nervous system, is suffering? The results of its adoption in the cases I have seen, and in those of the analogous diseases, accord with the after-death phenomena, in throwing serious doubts on the propriety of the measure, at least to the extent, and at the time at which it was employed, *although the blood was inflamed.* Experience, however, does not conclusively show the impropriety of a general bleeding, for the bleeding has usually been profuse. An abstraction of four ounces of blood I have known to be useful, when the loss of twenty ounces would have been injurious. In some cases, however, of this form, local bleeding and *extensive dry cupping* appear most proper; and leeches would often be more successful than they have been, if the blood vessels *directly communicating* with the seat of the local disease were depleted. Leeches *on the abdomen*, where only they are usually placed, could not act so powerfully in *metritis mucosa* as in inflammation of the parietal peritoneum; yet how seldom do we employ our knowledge of the seat of a disease and of the anatomy of the blood vessels in directing our local bleeding.

(b.) *Purgatives.*—The sixth case shows the sad consequence of costiveness after parturition; and, on the other hand, a violent purgative seemed in one instance to induce severe intestinal peritonitis; and there is reason from analogy to suspect that where an animal poison was in the system, purgatives have favored the rise of an erysipelatous disease of the intestines. The conditions of the special case will regulate their use; when the stomach and intestines were inflamed, a violent purge has done harm; and half an ounce of turpentine in an irritable body has excited gastritis. When the inflammation was confined to the uterus, free purging has had the most delightful effects; the aperient salines with colchicum, and mercury with chalk, antimonial powder and ipecacuanha, I have found, in these cases, act freely and safely on the bowels. In the *typhoid* variety of the disease, the best purgative is castor oil with turpentine, and *clysters* of the same medicine, and also of *calomel with soda*, in doses of half a drachm, of each.

(c.) *Sedative Diuretics.*—Digitalis, with the alkaline salines, given largely in many cases, did no good; although analogy appeared to recommend them. Colchicum I have found superior to the former, when the skin is hot and the pulse sharp. *Turpentine* appears to me to require more caution and discrimination in its use than it has at all times received; from its benefit in some *hospital* cases, it has been largely adopted in private practice; and in high-blooded irritable patients, and in the synochal form of the disease, I have known it do mischief. In one case it excited severe spasm of the diaphragm and abdominal muscles. No one thinks of using it in phlegmonous erysipelas; why should it be used indiscriminately in puerperal fever? In the more sthenic form of the disease, after the acute inflammation has relieved itself by serous effusion, this medicine may safely, perhaps, be used; and in the *typhoid* cases, no medicine has been more successful. There is nothing mysterious or *specific* in its operation in puerperal fever; it is only beneficial in this disease, in a state analogous to that of other diseases in which it has been efficacious.

(d.) *Mercury.*—Its high value in all the forms of the disease, is established by abundant evidence. The best mode of using it remains to be shown by *exact* experiments. The utility of *inunction* admits of little doubt; is not inserting it into the vagina worth a trial? The black-wash of calomel and *soda*, in cases with diarrhœa, is, I believe valuable. It is a favorite practice to give ten grains of calomel at first, and to repeat doses of three grains; I believe the smaller doses, at very short intervals, are better. Theory would reject it in the putrid form of the disease; but experience confirms its value.

(e.) *Narcotics.*—I would submit a doubt of their value, even when combined with mercury or antimony. Opium has done harm, and is, I believe, on a sound theory, to be condemned, except in the case of a very rapid pulse, with a moist tongue, a perspiring skin, and watery stools. In common phlegmasial inflammation, in child-bed, I have used it, and the salts of morphia, with the best effect, after bleeding; but in the puerperal fever it easily *narcotises* the patient, and adds to the prostration of the nervous power.

(f.) *Stimulants.*—In the stage of collapse in all cases and earlier in the typhoid form, they must be given to save the reputation of the practitioner, if the patient cannot be saved; they have been used *injuriously early* in the more sthenic variety of the disease. I have reason to believe that *chlorate of potass and ammonia* would, in some cases, be the best stimulant.

(g.) *Tonics* are not recommended by direct experience, but they are by the analogy of *putrid erysipelas.* Both analogy and empiricism *condemn the early use* of bark or quinine. The *diacetate of lead* is a sedative tonic which my experience in the case of *after flooding*, and the analogy of other putrid diseases, combine to recommend to further trial.

(h.) Of *local means*, emollient poultices, or hot moist bran, or boiled chamomile flowers, with vinegar of colchicum and tincture of henbane in them, I have found serviceable. In some cases more restringent applications, as dry heat, or hot flannels with alcohol sprinkled on them, or *hot salt* and mustard flour, may be preferred.

In conclusion, I would suggest that *new means* are not required; we are not to search for *specifics*, but to aim at a more distinguishing and judicious use of known remedies.—*Prov. Med. Journal.*

